# Design and Refinement of CyberCell^TM^, a Virtual Reality Health Education Program for Adolescents and Young Adults with Sickle Cell Disease

**DOI:** 10.1177/29941520251374284

**Published:** 2025-09-03

**Authors:** Aimee K. Hildenbrand, Melanie Pitone, Benjamin Bear, Corinna L. Schultz, Sherman Finch, Danielle Morley, Stephanie Hubbard, Alyssa Decino, Melissa A. Alderfer, Lori E. Crosby, Fredric Freeman

**Affiliations:** ^1^ Center for Healthcare Delivery Science, Nemours Children’s Health, Wilmington, Delaware, USA.; ^2^ Department of Pediatrics, Sidney Kimmel Medical College, Thomas Jefferson University, Philadelphia, Pennsylvania, USA.; ^3^ Division of Behavioral Health, Nemours Children’s Hospital Delaware, Wilmington, Delaware, USA.; ^4^ OnXR, Inc., Westminster, Maryland, USA.; ^5^ Lisa Dean Moseley Foundation Institute for Cancer and Blood Disorders, Nemours Children’s Health, Wilmington, Delaware, USA.; ^6^ College of Art and Media, Sam Houston State University, Huntsville, Texas, USA.; ^7^ Division of Behavioral Medicine and Clinical Psychology, Cincinnati Children’s Hospital Medical Center, Cincinnati, Ohio, USA.; ^8^ Department of Pediatrics, University of Cincinnati College of Medicine, Cincinnati, Ohio, USA.

**Keywords:** virtual reality, patient education, usability, immersive, sickle cell disease, shared decision-making

## Abstract

For people living with sickle cell disease (SCD), the risk for disease complications and premature death rises sharply in adolescence and young adulthood, when chronic organ damage is compounded by reduced preventive care. To make informed health care decisions, adolescents and young adults (AYAs) require effective education about their health condition and treatment options. We aimed to systematically design and develop a user-centered, evidence-based virtual reality (VR) health education program to optimize the use of SCD therapies among AYAs with SCD. We engaged a diverse team including AYAs with SCD and their caregivers, multidisciplinary SCD clinicians and researchers, patient education specialists, and health technology developers. Stakeholder meetings and codesign workshops were used to evaluate end-user and implementer needs, develop low- and high-fidelity prototypes of the VR program, and draft an implementation plan. Subsequently, iterative cycles of usability testing were conducted with 17 AYAs with SCD (*M* = 16.82 years, 59% male, 94% Black) and six SCD clinicians (83% female, 50% Black) to assess technical and functional reliability and to refine the implementation plan. Rapid assessment procedures were used to synthesize think-aloud field notes and qualitative interview data into a summary matrix of potential prototype modifications. Through these iterative, user-centered design processes, our team developed and refined CyberCell^TM^, a VR health education program for AYAs with SCD. CyberCell provides immersive, interactive, and evidence-based education about SCD and treatment options, with the goal of facilitating deeper learning and increasing motivation for health behavior change. Think-aloud procedures revealed various challenges with wayfinding and interaction in early prototype versions, which were addressed to improve user experience. Interviews demonstrated satisfaction with CyberCell and potential positive impacts on patient–provider communication and patient health behaviors. Future research will evaluate the feasibility and effectiveness of CyberCell on enhancing the reach and uptake of disease-modifying therapies, thereby improving health for AYAs with SCD.

Sickle cell disease (SCD) is a group of genetic disorders resulting in rigid, abnormally adhesive, and dehydrated red blood cells. Individuals with SCD can experience severe complications, including anemia, pain, stroke, and organ injury,^1^ which contribute to poor quality of life^2,3^ and early mortality.^1^ Risk for disease complications and premature death rises sharply in adolescence and young adulthood,^4,5^ when chronic organ damage is compounded by inconsistent follow-up and reduced preventive care.^6,7^

Safe and effective therapies are available for individuals with SCD, but pervasive research-to-practice gaps limit their reach. Hydroxyurea (HU) is the gold standard treatment for SCD.^1^ It reduces complications,^8–15^ health care utilization, and costs,^16,17^ and it improves quality of life,^18^ growth,^19^ and survival.^20,21^ Despite decades of strong evidence for HU,^1^ uptake has been slow.^22,23^ Recent data indicate that only about 25% of individuals with SCD are prescribed HU.^23^ Suboptimal HU use is especially notable among adolescents and young adults (AYAs), who have the fewest days supplied, longest breaks, and poorest adherence of all age groups.^24–26^ Barriers to HU utilization are multi-factorial.^27,28^ AYAs report lack of involvement in decision-making, inadequate knowledge, and doubts about efficacy and safety.^29–33^ Providers may not offer HU due to biased assumptions about patient adherence.^27,34^ These challenges are compounded by structural and social determinants of health obstacles to care that are prevalent in this population (e.g., limited transportation, low health literacy).^27^ Similar challenges limit the use of newer therapies that have been approved by the FDA in recent years (i.e., L-glutamine, crizanlizumab),^35–37^ with current uptake estimated at <5% of the population.^23^

To make informed health care decisions, AYAs require effective education about their health condition and available treatment options. Unfortunately, existing SCD education strategies are not user-centered, grounded in health promotion theory, or designed for dissemination.^38–40^ For example, pharmacy handouts on HU rarely identify SCD as an indication for use, often contain inaccuracies related to long-term cancer risk, and have an average reading level two grades higher than recommended for patient education.^41^

Virtual reality (VR) is a promising approach to delivering health education for AYAs with SCD. Given the superiority of immersive, “learning-by-doing” approaches over traditional instruction,^42,43^ VR is gaining momentum as a patient education tool in health care.^44^ VR has demonstrated efficacy in improving health knowledge and promoting adoption of preventive behaviors in adult patients and caregivers.^45–49^ Very few studies have used VR for patient education in pediatrics, demonstrating high satisfaction among youth undergoing chest radiography^50^ and endoscopic procedures.^51^ However, VR-based instruction is widely employed in K-12 education, with strong evidence of enhancing knowledge and skills.^52^ As such, VR education represents a promising avenue for enhancing AYAs’ knowledge and motivation to use disease-modifying therapies best aligned with their values and preferences.

Guided by the Accelerated Creation-to-Sustainment (ACTS) model,^53^ we aimed to systematically codesign, develop, and refine a user-centered, evidence-based VR health education program to optimize use of therapies among AYAs with SCD. The ACTS model outlines three stages to rapidly move from initial project conceptualization to a sustainable technology-enabled service: Create, Trial, and Sustainment.^53^ This paper presents our efforts to complete the Create stage of the model, which focuses on employing user-centered design^54^ to engage with key stakeholders in developing a minimally viable technology (i.e., functional, free of major usability issues, and safe) and implementation plan that is ready for first deployment and can be further optimized in the next stage. We also report on the acceptability and usability of the program and explore implementation barriers and facilitators.

## Materials and Methods

### Phase 1: Design and develop

#### Ethical considerations

All study procedures (Phases 1 and 2) were reviewed and approved by the Nemours Children’s Health Institutional Review Board (#1600953). No research participants were enrolled and no study data were collected during Phase 1, which involved codesign workshops and meetings with our stakeholder workgroup (i.e., members of the project team). This study is reported in accordance with guidance for reporting intervention development studies in health research (GUIDED).^55^

#### Stakeholder workgroup

We assembled a diverse team including AYAs with SCD and their caregivers, multidisciplinary SCD clinicians and researchers, patient education specialists, and health technology developers. Most technology-enhanced interventions evaluated in clinical trials were developed with limited input from end users,^53^ contributing to pervasive challenges with high attrition.^56^ In contrast, we sought to involve end users (AYAs with SCD) and implementers (SCD health care providers) in every stage of intervention design and development to ensure that our VR prototype was relevant, appealing, practical, and intuitive. Stakeholders were also engaged in all aspects of study planning and execution to enhance alignment of this research with the priorities, preferences, and needs of end users.^57^

#### Procedures

Consistent with the Create stage of the ACTS model,^53^ we first focused on obtaining information from our stakeholder workgroup via bimonthly virtual meetings and two half-day codesign workshops (i.e., intensive ideation and design sessions) at Nemours Children’s Hospital Delaware (NCHD), with remote participation options for those who could not attend in-person. NCHD is a children’s hospital in the mid-Atlantic region of the United States. During initial meetings, we collaboratively brainstormed end-user and implementer needs and preferences for a VR health education program, linked those requirements to proposed VR prototype content and features, and created and obtained feedback on low-fidelity prototypes (e.g., storyboards, basic 3D models). These meetings informed the development of a higher-fidelity version, which stakeholders tested during the first codesign workshop. In addition to providing input on the VR prototype, stakeholders engaged in a free listing activity to generate ideas for additional content and features. Subsequent meetings involved further specification of more fully functioning, higher-fidelity prototypes. During the second codesign workshop, members of the stakeholder workgroup tested a high-fidelity version of the VR program and collaborated to draft an initial implementation plan including strategies, timeframe, milestones, and performance measures in preparation for a future trial.^58^

#### CyberCell^TM^: A sickle cell VR experience

Through these iterative, user-centered design processes, our team developed CyberCell, a VR health education program for AYAs with SCD. CyberCell provides immersive, interactive, and evidence-based education about SCD and treatment options, with the goal of facilitating deeper learning and increasing motivation for health behavior change. CyberCell enables users to visualize and interact with healthy and sickled red blood cells, observe health complications associated with sickling, and explore how disease-modifying therapies (i.e., HU and crizanlizumab) impact red blood cell functioning within a simulated three-dimensional blood vessel environment (see Fig. 1).

**FIG. 1.**
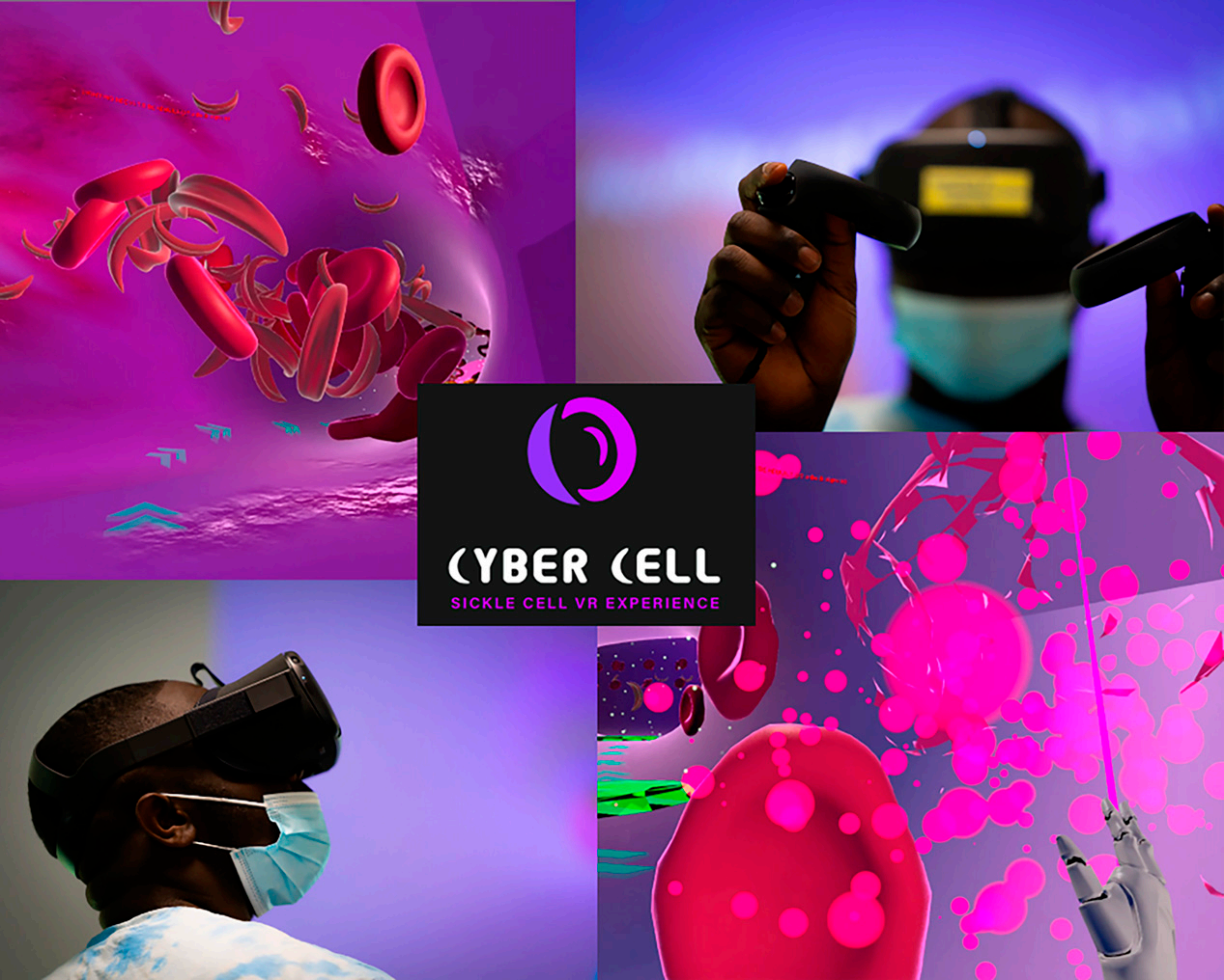
CyberCell: A sickle cell VR experience. Member of our stakeholder workgroup (young adult with SCD) using CyberCell (top right and bottom left) and snapshots of the user’s perspective within CyberCell (top left and bottom right). VR, virtual reality.

CyberCell content and features were informed by our prior research, which revealed that decisions about starting disease-modifying therapies are strongly shaped by patient knowledge and beliefs (e.g., understanding of how therapies work, perceived safety and efficacy of treatments, awareness of ongoing organ damage from untreated SCD).^30,59^ In addition, AYAs desired education about SCD and treatment options that is engaging, easy to understand, and offered in multiple formats (e.g., written, audio, visual), underscoring the need to transform and improve how health education is delivered for this population. CyberCell is also guided by the Health Belief Model (HBM),^60^ which posits that health behaviors (e.g., using a disease-modifying therapy) are influenced by perceived susceptibility and severity (e.g., of SCD-related complications) as well as perceived benefits (e.g., prevention or reduction of complications) and barriers (e.g., concerns about side effects). Educational programs informed by the HBM have demonstrated success in enhancing adoption of health behaviors.^61–65^

### Phase 2: Refine

#### Ethical considerations

Research team members contacted potential study participants (i.e., AYAs with SCD, hematology clinicians) via mail, phone, email, and/or during SCD clinic appointments at NCHD. Research team members explained the study to potential participants and reviewed the informed consent (and assent, for patients <18 years of age) documents in a private, quiet space in the hospital. Potential participants were encouraged to ask questions about the research and given time to decide about participating. Those who agreed to proceed with participating documented their consent/assent for the research project by signing the consent/assent form.

#### Procedures

Consistent with the ACTS model, we conducted a formal usability evaluation and intensive qualitative assessment to refine the VR prototype and implementation plan. Specifically, we recruited AYAs with SCD (ages 13–21 years) and hematology clinicians (who did not serve on our stakeholder workgroup) to participate in iterative cycles of usability testing to assess technical (i.e., software and hardware perform consistently) and functional reliability (i.e., users can perform the intended actions) and provide feedback on the draft implementation plan. Patients were contacted by mail, phone, email, and/or during a clinic appointment. Health care providers were contacted via email. Following assent/consent, usability testing sessions were conducted at NCHD. Participants used the VR prototype while “thinking aloud,” a well-established procedure to obtain *in vivo* feedback from end users.^66^ Field notes were used to document any technical difficulties and observations on user engagement. Next, using a semi-structured interview, we assessed perspectives on the VR prototype (e.g., ease of use, clarity, acceptability, recommendations for improvement) and its implementation (e.g., preferences for strategies to integrate the VR program into clinical care). Usability testing sessions were audio- and screen-recorded and transcribed. Participants received $25.

#### Participants

Patients were recruited using maximum variation purposeful sampling^67^ to ensure a diverse sample with regard to demographic and clinical characteristics (e.g., age, sex, socioeconomic status, disease severity) and extent of exposure to disease-modifying therapies. Youth were eligible to participate if they were: (a) of age 13–21 years, (b) diagnosed with SCD, and (c) receiving sickle cell care at NCHD. We also invited all NCHD providers who specialize in caring for youth with SCD to participate. English language fluency was a requirement for all participants. Youth and providers who reported history of seizures were excluded.

#### Measures

##### Demographic and clinical characteristics

Electronic health record review and a self-report survey were used to assess participant clinical and demographic characteristics.

##### Usability testing guides

Separate usability testing session guides were developed for patients and providers (see Supplementary Data). Participants were asked to use the VR prototype while verbalizing what they were thinking, looking at, doing, and feeling. The interview guide then prompted additional perspectives on the VR prototype (e.g., overall impressions, difficulties encountered, experience navigating the virtual environment) and implementation (e.g., ways to integrate the prototype into a typical clinic visit).

#### Data analysis

Participant demographic and clinical characteristics were summarized using descriptive statistics. Data analysis began once the first usability session was completed to inform real-time modifications to the VR prototype before subsequent testing cycles. Rapid assessment procedures (RAP)^68^ were used to synthesize think-aloud field notes and qualitative interview data into a summary matrix of potential prototype modifications. RAP is a team-based, highly efficient qualitative analytic approach^68–70^ that is commonly used in program planning and evaluation.^71,72^ Following RAP analysis, data on usability challenges and acceptability were summarized into broader themes via team discussion. Qualitative data on future implementation of the VR program within clinical settings were organized into barriers and facilitators using the Consolidated Framework for Implementation Research (CFIR),^73^ which synthesizes theories and empirical work on factors most likely to influence implementation of an innovation.

## Results

In total, we contacted 26 AYAs with SCD and 8 health care providers who specialize in caring for youth with SCD to invite them to participate in Phase 2. One AYA declined, 8 were interested but did not enroll due to time constraints, and 17 AYAs consented and participated. Two health care providers did not respond to the study invitation email, and six providers consented and enrolled. Most AYAs identified as male (59%), non-Hispanic/Latinx (88%), and Black or African American (94%; see Table 1). Most health care providers were female (83%) and non-Hispanic/Latinx (100%). Health care providers identified as Black or African American (50%), White (33%), and multiracial (17%).

**Table 1.** Participant Demographic Characteristics

Patient participants (*N* = 17)	Frequency (%)
Gender	
Female	7 (41.2)
Male	10 (58.8)
Race	
Black or African American	15 (88.2)
Multiracial (Black and White)	1 (5.9)
Not reported	1 (5.9)
Ethnicity	
Hispanic or Latinx	2 (11.8)
Non-Hispanic or Latinx	14 (82.4)
Not reported	1 (5.9)
Sickle cell disease genotype	
SS	15 (88.2)
SC	2 (11.8)
	M (SD), range
Age (years)	16.82 (2.56), 13–21
Health care provider participants (*N* = 6)	Frequency (%)
Gender	
Female	5 (83.3)
Male	1 (16.7)
Race	
Black or African American	3 (50.0)
White	2 (33.3)
Multiracial (Asian and White)	1 (16.7)
Ethnicity	
Hispanic or Latinx	0 (0)
Non-Hispanic or Latinx	6 (100)
Discipline	
Nurse practitioner	2 (33.4)
Physician	2 (33.4)
Psychologist	1 (16.7)
Social worker	1 (16.7)
	M (SD), range
Years in practice	13.92 (9.97), 5–30

### Usability

Think-aloud procedures revealed difficulties related to wayfinding and navigation within the three-dimensional environment and completion of more complex, interactive user actions in early prototype versions. For instance, some users had difficulty understanding when and how to move forward through the experience, as well as how to interact with blood cells (e.g., the grabbing function). In addition, the VR experience was initially intended to be used by participants while standing, but some users preferred to sit or were unable to stand due to receiving treatments (e.g., blood transfusion) that required them to remain in a hospital bed. Modifications were made iteratively to address these challenges (see Fig. 2), including enhancements to the instructions users receive both before and during the VR experience, the addition of audio and visual wayfinding markers and navigational cues, simplifying 3D models, incorporating visual design elements such as color and contrast to aid in drawing user attention to directional cues, and enabling multiple options for viewing the VR environment (e.g., physically moving one’s head and body vs. use of the controller’s joystick). No adverse events were reported during usability testing of this VR experience.

**FIG. 2.**
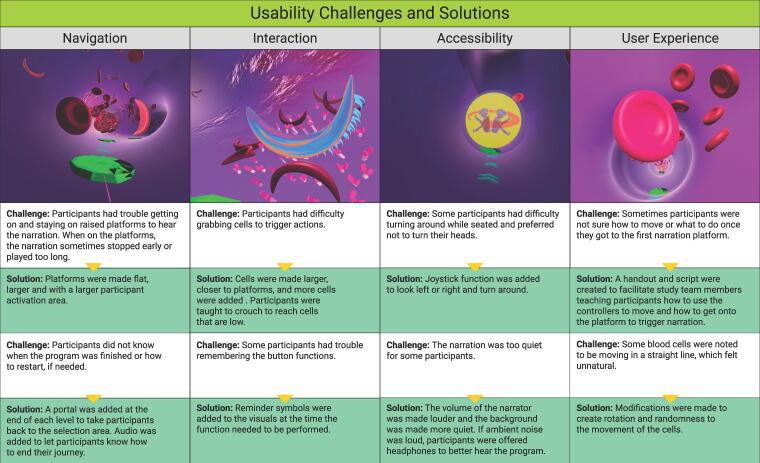
Usability challenges and solutions. Usability issues that arose during think-aloud testing and corresponding solutions implemented.

### Acceptability

Qualitative interviews revealed several themes related to acceptability of CyberCell: (1) CyberCell is an engaging, immersive, and interactive way to learn about SCD and treatment options; (2) deeper learning enhances AYAs’ motivation for health behavior change (i.e., medication initiation or adherence); and (3) AYAs with SCD and their health care providers report high satisfaction with VR as an approach to educating patients and facilitating communication between patients and providers (see Table 2 for illustrative quotes).

**Table 2.** Acceptability Themes Identified and Illustrative Quotes

Theme	Illustrative quotes
CyberCell is an engaging, immersive, and interactive way to learn about SCD and treatment options	“It’s more in-depth because its hands-on. Instead of someone telling you, you really get to see what actually happens … You get a deeper understanding.” (20-year-old male with SCD) “Showing what happens inside the blood vessel is … because you can’t really see inside your blood vessel, so it’s really nice to see with your own eyes … Like, for criz for example. I didn’t know it made the blood cells bounce off [blood vessel walls].” (19-year-old male with SCD) “Not only does it give a nice overview of what the medicines do, but even bigger than that, it gives a beautiful picture of how sickle cell works inside the blood vessels. And I like that it shows not just the sickle cells clogging up, but it shows the vessel damage. It shows hemolysis and explains what that is. And it’s very simple.” (Pediatric Hematologist)
Deeper learning enhances motivation for health behavior change	“I did not know there was anything else to learn about sickle cell. I thought I knew everything. I learned what hydroxyurea does, that one was an enlightening revelation. I didn’t know hydroxyurea was that impressive … now I’ll take it seriously.” (20-year-old male with SCD) “…You don’t how it actually works, you’re just going based off what somebody’s telling you. So, seeing it—you get to see like, ‘Oh, this is how it’s benefitting me, maybe I should take it more, or learn about it more for myself.’ It’s just different when somebody’s just telling you versus you actually looking into it …. I feel like it’s important to take [hydroxyurea] more than I actually do.” (19-year-old female with SCD) “I think [CyberCell would help] if you’re talking to somebody for the first time about the medicines. I think if you’re trying to convince somebody that’s not compliant why they should be compliant. Somebody who was really little when they started a drug and maybe doesn’t know how it works, and you feel like they’re old enough to understand it [now].” (Pediatric Hematologist)
AYAs with SCD and their health care providers are highly satisfied with VR as an approach to educate patients and facilitate patient-provider communication	“It blew me away because I thought it would be just like, “Okay, we got new medicines for you to go through. That’s all you can do.” I didn’t think we was going to be able to actually interact with the cells. So, I was really, really excited about that part, and I think other people would like it, too … A lot of people don’t like learning, but [VR] makes it fun.” (18-year-old female with SCD) “[VR] is more fun for kids especially. We’re seeing information from doctors just talking. It is kind of boring. This is more entertaining.” (16-year-old female with SCD) “For patients and caregivers whose health literacy is not as high, it’s easier to really just listen to it and see it rather than having to read something.” (Pediatric Psychologist)

#### Theme 1: CyberCell is an engaging, immersive, and interactive way to learn about SCD and treatment options

Most AYAs and health care providers described high satisfaction with CyberCell as a health education program because of its emphasis on engagement, immersion, and interactivity. For example, a young adult commented:

“I liked that [CyberCell] gave you a different way to learn things. Because a lot of people don’t understand something because they’re more of a kinesthetic learner, and that’s as hands-on as you’re going to get … I learned about cell docking stations. And I learned that sickle cells aren’t just like cells that are sickle shaped. That they can go from whole to sickled to whole again.” (18-year-old female with SCD)

Similarly, a health care provider noted:

“It’s one thing to talk about sickle cells, but seeing the cells and then seeing what these medications actually do … just reaching out and touching things made it fun … it’s one thing to talk about vessels like it’s an abstract concept. Actually seeing [sickle cells] stuck together, to be able to provide that visual representation, is amazing.” (Pediatric Hematology Nurse Practitioner)

#### Theme 2: Deeper learning enhances AYAs’ motivation for health behavior change (i.e., medication initiation or adherence)

Participants also discussed that by facilitating enhanced understanding of SCD and treatment options, CyberCell may promote increased motivation for adaptive health behavior change among AYAs with SCD. For instance, when reflecting on her experience using CyberCell, an adolescent noted:

“When it talked about hydroxyurea, what it does and how it can help, it was interesting because I didn’t really know what it did. I take the medicine, but I didn’t really know what it did. It makes you want to take the medication because you see what it does with your own eyes.” (14-year-old female with SCD)

#### Theme 3: AYAs with SCD and their health care providers report high satisfaction with VR as an approach to educating patients and facilitating communication between patients and providers

Finally, AYAs with SCD and their health care providers described enthusiasm around the use of VR to deliver patient education and promote meaningful conversation between patients and providers. Considering how CyberCell compares to existing patient education approaches, one clinician reported:

“[VR] is more exciting and interactive and gives people choices… it allows them to take control about what they hear about. And, if they want to go back and look at something again, then they don’t have to worry about asking to repeat or circle back for clarification… If you’re talking about a new medicine or introducing a new therapy to somebody, [CyberCell] might be a way to help them understand why or what it does… We should think about it as a way to engage our patients and open a conversation.” (Lifespan Hematologist)

### Implementation barriers and facilitators

Finally, qualitative interviews with AYAs and health care providers highlighted several potential facilitators and barriers to implementing CyberCell as a component of sickle cell care (see Table 3). Barriers and facilitators fell within two CFIR domains: (1) Innovation and (2) Inner Setting. With regard to CyberCell itself (i.e., the innovation), AYAs and health care providers identified several perceived advantages relative to existing patient education approaches (e.g., easier to understand and remember, more engaging and interactive). For instance, one young adult with SCD commented:

**Table 3.** Implementation Barriers and Facilitators Identified and Illustrative Quotes

CFIR construct	Potential barriers and facilitators	Illustrative quotes
Domain: Innovation		
Innovation relative advantage	Facilitator: CyberCell offers advantages over traditional patient education modalities	“You remember [CyberCell] more than you would a doctor talking. When a doctor gives a speech, it’s hard to understand. When you see something, it has a different effect… you remember a lot of the information.” (20-year-old male with SCD) “[Doctors] just talk to me. They’ll give me booklets and pamphlets. I’m going to be honest, I don’t think I ever read the pamphlets. The VR is more fun… [it is] a fun way to explore while also learning about sickle cell.” (18-year-old female with SCD) “[CyberCell] is a way to interpret or translate some of the things that clinicians talk about into language or concepts that might be more easily accessible to patients or help them learn in a way that’s different than just listening or reading… It’s more interactive and entertaining. And, I think it is probably a lower health literacy level or more approachable than what we as clinicians tend to say to people.” (Lifespan Hematologist) “[CyberCell] is just much more visual and much more fun for younger people. It gets them involved in it instead of just passive listening.” (Pediatric Hematologist)
Innovation Complexity	Barrier: Learning how to use and administer CyberCell can be complex	“I think you got to get past the point of learning what you’re doing… For someone that doesn’t [have experience using VR], I think you’ll spend more time trying to teach them what to do [when using CyberCell].” (Social Worker) “It might be helpful to just give people a quick minute or two to sort of test it out within the VR thing. Testing it out to see if they have any questions or difficulties before it starts. Maybe that could be helpful, a short test run. I think once that’s done, I think it’ll be fine for most people.” (Pediatric Psychologist)
CFIR Domain: Inner Setting		
Compatibility	Facilitator: CyberCell is compatible with clinical workflows, systems, and processes	“It’s not too long, but it’s long enough.” (19-year-old male with SCD) “Before the doctor comes in say, ‘Hey, we did your weight, and now you’re waiting for the doctor. You have 15–20 minutes. You can [use CyberCell], and then talk to the doctor.’ And then, it may bring up questions to ask [the provider].” (Social Worker) “The best time [to use CyberCell] would be if patients were in the waiting room [or] if they’re here for those longer days, like hem/pulm or hem/optho [clinic], maybe in between seeing providers… We could [also] use it on the inpatient side [when] we’re giving them information about [a therapy that] we’re advocating for.” (Pediatric Hematology Nurse Practitioner) “[At] a check-up, the routine [could be]: you sit down, your doctor comes in and sits next to you, they give you a headset, and then they explain it. That would be great. That would be the perfect balance of these two elements…You don’t want to take away the human connection. You don’t want to just give me the headset and I sit here and watch and then go home.” (20-year-old male with SCD)
	Barrier: CyberCell is not accessible to patients who do not speak English	“Certainly, people who don’t speak English wouldn’t be able to access it in its current form.” (Lifespan Hematologist)
Available Resources	Barrier: Resource constraints (i.e., time, staff) are likely challenges to using CyberCell in clinical settings	“The providers are moving from patient to patient and wanting to get through it. So, finding that time, when would they be able to do the VR? That will be the biggest barrier.” (Pediatric Hematology Nurse Practitioner) “Time, I don’t think would be a big barrier because again, it didn’t seem to take that much time. Although, I know our families are sometimes eager to just get the visit over with.” (Pediatric Psychologist) “Once it’s not a research thing, who would support it? That’s really—I don’t know. I would like to say that I’d be willing, but I just don’t know that I’d be able to all the time. I also don’t know that the other providers would, for different reasons. I wonder if it could be something that could be a volunteer role, but they’d have to be savvy with this kind of thing. Dang, we need more staff.” (Pediatric Hematology Nurse Practitioner)

CFIR, Consolidated Framework for Implementation Research.

“A lot of informational things, they’re usually very bland. You never really see something eye-catching. They’re usually just a pamphlet, something you flip through a couple times. This [CyberCell] is interactive… This is definitely one of the easiest ways – and interactive and fun ways – to get to learn about sickle cell… it’s really a game changer in explaining medicines and breaking down treatments. This is really a game changer.” (19-year-old male with SCD)

On the other hand, several clinicians described that the complexity of learning how to use CyberCell is an important barrier to implementation in busy clinical settings, with one noting:

“The learning curve [to administer CyberCell] is the biggest [barrier]. The younger population may be able to catch on to it quicker, but just getting through that part of it, as opposed to, it’s easy to talk to someone and hand out pamphlets… Initially, I’m like, ‘What am I taking in if I’m trying to figure out how to navigate the system? Am I learning anything, or am I just learning how to navigate the VR?’ Which is what I felt like my first couple minutes was, me learning the VR.” (Pediatric Hematology Nurse Practitioner)

Within the Inner Setting domain, participants discussed that CyberCell is compatible with existing clinical workflows, systems, and processes. For example, many participants noted that CyberCell is appropriate in length, with one pediatric hematologist commenting: “[The length was] perfect. Not too long, not too short.” Others described how CyberCell could be implemented across different clinical settings, including outpatient clinics, infusion centers, and inpatient hospital rooms:

“Before I get the blood drawn, I have some free time. Or, after I get finished talking to the doctor. [AYAs could use CyberCell] in here [infusion center] because you’ll have space to stand up and move around, it won’t be in anyone’s way.” (21-year-old male with SCD)

“When you’re admitted… whenever they’ve settled in, they have a bit of free time… when I was admitted, if I wasn’t in pain, I was pretty bored.” (18-year-old female with SCD)

However, one clinician noted an important compatibility barrier for clinics that serve individuals with SCD who do not speak English, in that CyberCell is currently available only in English. In addition, several health care providers noted resource constraints (i.e., time, staff) that may hinder use of CyberCell in clinical settings:

“Somebody actually has to take the time to set it up, put it on the person, and tell them how to do it, and stand there. If you are going to put it in a provider visit, that’s 15 minutes… [It may be] better to have somebody else other than the provider that’s doing the visit to [administer CyberCell] because I think you’re going to have trouble with provider time.” (Pediatric Hematologist)

## Discussion

Overall, findings suggest high usability and acceptability of CyberCell among AYAs with SCD and their health care providers. Think-aloud testing revealed various challenges that were addressed to improve user experience. Qualitative interviews with patients and providers demonstrated satisfaction with VR as an approach to provide hands-on learning about SCD and treatment options, and potential benefits of this improved knowledge on patient–provider communication and patient health behaviors. Together, think-aloud procedures and interviews also provided key insights about implementation that will inform additional CyberCell modifications to facilitate future dissemination and sustainment of this VR health education program.

Our formative usability testing, comprised of think-aloud procedures and qualitative interviews, played a critical role in developing and refining CyberCell. Based on observations and feedback received during usability testing, we implemented various design changes to address challenges related to wayfinding and interaction between the user and elements of the virtual blood vessel environment. These changes were made iteratively, such that updated prototypes were tested on subsequent usability testing participants. While successful in identifying nearly all usability issues that users were likely to experience, this method required many rounds of prototype modification. In the future, it may be more efficient to batch observations and feedback from usability testing and perform a predetermined number of planned rounds of modification. In addition, the use of automated tools to obtain objective measures of usability (e.g., head, hand, or eye tracking, speed of task performance)^74^ might supplement the subjective data obtained through think-aloud procedures and interviews.

While we did not formally evaluate the impact of CyberCell on knowledge or behavior in this usability study, our qualitative data are consistent with a recent meta-analysis, which found that immersive VR learning approaches are more effective for both knowledge and skill development than nonimmersive approaches, especially for children and adolescents and in the area of science education.^42^ Our findings are also consistent with research demonstrating that digital health technologies (broadly defined) that support disease self-management are highly acceptable among individuals with SCD.^75^ Importantly, our study also adds to a small yet growing body of evidence supporting the role of VR in improving health education and healthy behaviors among individuals with chronic illness.^76^

Several aspects of CyberCell were identified as helpful for future large-scale dissemination. Prior work suggests that health care providers are more likely to adopt VR in clinical practice when there is clear value added by this technology.^77^ In this study, both patients and providers noted that CyberCell is an effective way to engage AYAs in their care and facilitate patient–provider communication. They also reported that CyberCell explains complex medical information in a way that is easily understood by AYAs. The program was flexible enough to be implemented across clinical settings (e.g., outpatient clinic, infusion center, inpatient hospital room). While patients and providers found CyberCell appropriate in length, time constraints in busy clinical settings where youth with SCD receive care were identified as a potential barrier. In addition, participants noted that superusers or in-experience tutorials may be needed to assist patients with less prior exposure to or self-efficacy with using VR. These findings align with existing research, with a recent scoping review highlighting that insufficient practical resources including time, technical support, equipment, and physical space are frequently identified barriers to implementation of VR in health care.^77^ To improve ease of deployment, we are building an in-experience tutorial (i.e., instructions for how to use CyberCell and demo space to practice skills). We also plan to explore opportunities to translate CyberCell into other languages, deploy remote synchronized VR experiences between multiple users (e.g., patient and caregivers or providers), and distribute content to devices remotely to improve accessibility, implementation, and broad dissemination.

Several limitations should be noted and considered. This study was conducted at a single pediatric SCD center, and thus, findings may not generalize to all settings in which AYAs with SCD receive care within the United States and globally. While the evaluation of feasibility and effectiveness was outside of the scope of this study, these data will be important to collect in future research. Future work will also explore additional content for patients, adaptations for other users (e.g., caregivers, health care trainees, and providers), language and cultural adaptation, and investigation of methods to support scaling and spreading this program to the broader sickle cell community.

This study supports the use of VR to provide engaging and interactive education to AYAs with SCD about their condition and treatment options. We are currently conducting a pilot pragmatic trial to evaluate the feasibility and preliminary effectiveness of CyberCell as one component of a broader shared decision-making toolkit for AYAs with SCD. Ultimately, we aim to produce a user-centered, acceptable, feasible, effective, and sustainable VR experience that can facilitate education, empowerment, and self-management among AYAs living with SCD.

## Abbreviations Used

ACTS: Accelerated creation-to-sustainment
AYAs: Adolescents and young adults
CFIR: Consolidated framework for implementation research
GUIDED: Guidance for the reporting of intervention development
HBM: Health belief model
HU: Hydroxyurea
NCHD: Nemours children's hospital delaware
RAP: Rapid assessment procedures
SCD: Sickle cell disease
VR: Virtual reality
