# Supplementary material for: Design and Refinement of CyberCellTM, a Virtual Reality Health Education Program for Adolescents and Young Adults with Sickle Cell Disease
Source: J Med Ext Real. 2025 Sep 3;2(1):190–202. doi: 10.1177/29941520251374284 (PMC12516198; doi:10.1177/29941520251374284)
Supplement: Supplementary file 1 [file 29941520251374284_supp_data.docx]

**SEMI-STRUCTURED INTERVIEW GUIDE (PATIENT VERSION)**

A. **Introduction**

We want to hear what you think about a virtual reality education program designed for teens and young adults with sickle cell disease. First, you’ll get to try out the VR program. Afterward, I will ask you questions about your experience and how you think the tool can be used in sickle cell clinic.

B. **Think Aloud**

For this part, I am going to ask you to think aloud as you use the VR program. What I mean by think aloud is that I want you to tell me everything you are thinking from the time you put on the VR headset to when you take it off. Try to talk out loud constantly. Do not try to plan out what you say, instead say what immediately comes to mind. Do you have any questions before we start?

*Prompts while participant is using CyberCell:*

a. What do you see?
b. What are you doing?
c. Tell me more about that.

C. **Teach Back**
   a. We want to make sure that everything in the VR program is easy to understand. In your own words, can you describe what you learned today?

*Prompts:*

i. What did you learn today that was new?
ii. Did any of the information surprise you?

D. **Feedback on CyberCell**

For this part, I am going to ask you questions about the VR program and how you think it could be used with teens and young adults with sickle cell disease. There are no right or wrong answers to these questions. If you want to skip a question or if you want to stop answering questions, you can. If anything is confusing, please let me know so that I can explain it better or repeat the question.

1. **Perspectives on CyberCell:**
   a. Before you tried the VR experience, what did you hope it would be like?
      i. How did the experience compare to your expectations?
   b. Overall, what did you think about the VR program?
      i. What did you like best? Why? [prompts: what parts did you find most helpful? Most interesting?]
      ii. What did you dislike/ like the least? Why?
   c. What parts of the VR experience grabbed your attention?
      i. What parts did you want to spend more time on?
   d. What parts of the VR experience were difficult or frustrating?
      i. What was confusing, unclear, or hard to understand?
      ii. What made the VR program difficult to use?
      iii. Were there any moments where you wanted to stop/take off the headset? Tell me more.
   e. What did you think about the length of the program?
   f. What was your experience with moving around in the VR space?

*Prompts:*
i. Was it clear where to go next?
ii. Did you understand how to move around using the controllers?
iii. Was it clear how to interact with the cells?
iv. What could help you move around more easily within the VR program?

g. What made you choose to learn about [medicine(s) they selected]?
   i. [If participant went through all levels]: Was the way each medicine was presented similar/ equal/ fair?
   ii. Was there anything about the VR program that made you think the program designers prefer one medicine over another?
h. If you were designing this, what would you change or add?

2. **Integrating the tool into sickle cell disease clinical care**
   a. How does the VR program compare to ways that you usually learn about sickle cell disease and treatments?
      i. What are advantages of VR compared to [*usual learning methods*]?
      ii. What are disadvantages of VR compared to [*usual learning methods*]?
   b. Thinking about your typical sickle cell clinic appointment, where would the VR program fit in?
      i. Where should the VR program be used?
      ii. When should the VR program be used (e.g., when first learning about a medicine)?
   c. How should the VR tool be introduced to patients and families?
      i. Who should introduce it?
      ii. What should patients know about the VR program before using it?
   d. Any other thoughts or feedback about the VR program?

*At the end of the interview, thank participant for their time and sharing their experiences. End audio recording.*

**SEMI-STRUCTURED INTERVIEW GUIDE (HEALTHCARE PROVIDER VERSION)**

A. **Introduction**

We want to hear what you think about a virtual reality education program designed for teens and young adults with sickle cell disease. First, you’ll get to try out the VR program. Afterward, I will ask you questions about your experience and how you think the tool can be used in sickle cell clinic.

B. **Think Aloud**

For this part, I am going to ask you to think aloud as you use the VR program. What I mean by think aloud is that I want you to tell me everything you are thinking from the time you put on the VR headset to when you take it off. Try to talk out loud constantly. Do not try to plan out what you say, instead say what immediately comes to mind. Do you have any questions before we start?

*Prompts while participant is using CyberCell:*

a. What do you see?
b. What are you doing?
c. Tell me more about that.

C. **Feedback on CyberCell**

For this part, I am going to ask you questions about the VR program and how you think it could be used with teens and young adults with sickle cell disease. There are no right or wrong answers to these questions. If you want to skip a question or if you want to stop answering questions, you can. If anything is confusing, please let me know so that I can explain it better or repeat the question.

1. **Perspectives on CyberCell:**
   a. Before you tried the VR experience, what did you hope it would be like?
      i. How did the experience compare to your expectations?
   b. Overall, what did you think about the VR program?
      i. What did you like best? Why? [prompts: what parts did you find most helpful? Most interesting?]
      ii. What did you dislike/ like the least? Why?
   c. What did you think about the length of the program?
   d. Was the way each medicine was presented similar/ equal/ fair?
      i. Was there anything about the VR program that made you think the program designers prefer one medicine over another?
   e. If you were designing this, what would you change or add?
2. **Integrating the VR program into sickle cell disease clinical care**
   a. How does the VR program compare to ways that you usually teach patients about sickle cell disease and treatments?
      i. What are advantages of VR compared to [*usual teaching methods*]?
      ii. What are disadvantages of VR compared to [*usual teaching methods*]?
   b. Which patients might benefit the most from using the VR program? (Examples: age, genotype, disease severity, learning style/ needs, eligible for/ making a decision about a treatment)
   c. Thinking about your typical sickle cell clinic appointment, where would the VR program fit in?
      i. Where should the VR program be used?
      ii. When should the VR program be used (e.g., when first learning about a medicine)?
   d. How should the VR tool be introduced to patients and families?
      i. Who should introduce it?
      ii. What should patients know before using the VR program? (Example: about sickle cell disease or a medicine, about the VR program itself)
      iii. Who should help the patient use the VR program? (In other words, who should facilitate or administer the VR program to the patient?)
   e. What barriers might get in the way of using the VR program in sickle cell clinic?
   f. Any other thoughts or feedback about the VR program?

*At the end of the interview, thank participant for their time and sharing their experiences. End audio recording.*
